# The assessment of psychological richness, meaning, and happiness with social media text data: Predictive accuracy and distinct behavioral correlates

**DOI:** 10.1371/journal.pone.0337649

**Published:** 2026-01-07

**Authors:** Cavan V. Bonner, Young-Min Cho, Fanyi Zhang, Louis Tay, Lyle Ungar, Sharath Chandra Guntuku

**Affiliations:** 1 Department of Psychology, University of Illinois at Urbana-Champaign, Champaign, Illinois, United States of America; 2 Department of Computer Science, University of Pennsylvania, Philadelphia, Pennsylvania, United States of America; 3 Department of Psychological Sciences, Purdue University, West Lafayette, Indiana, United States of America; De Montfort University, UNITED KINGDOM OF GREAT BRITAIN AND NORTHERN IRELAND

## Abstract

Assessing well-being with social media text data is a promising method, but besides hedonic well-being, little is known about whether additional well-being dimensions, such as psychological richness and eudaimonic well-being, can be predicted from such data. We compare the predictive accuracy for hedonic well-being, eudaimonic well-being, and the recently proposed construct of psychological richness in a large sample of Facebook users (*n* = 2,644), and find that the inclusion of language features incrementally improved model prediction accuracy beyond demographic features for psychological richness, but not for hedonic or eudaimonic well-being. Psychological richness had the lowest overall prediction accuracy (*r* = .21) followed by hedonic well-being (*r* = .27) and eudeomonic well-being (*r* = .29). The linguistic features associated with Psychological Richness were face valid, and in many instances the content and direction of the associations were unique to Psychological Richness, which provides discriminant validity evidence.

## Introduction

The health, performance, and social consequences of hedonic well-being (also known as subjective well-being [SWB]; e.g., [1,2]), have inspired efforts to monitor well-being at the national and international levels [3]. However, traditional representative survey approaches are limited by low response rates, perceived intrusiveness, high costs, and their inability to comprehensively monitor well-being over time [4]. Recent attempts to automatically predict well-being and other individual differences based on language features extracted from social media platforms have demonstrated that *language-based assessments* (LBAs) are a promising alternative to traditional surveys. Language features extracted from social media platforms such as Facebook and Twitter accurately predict self-reported SWB [5–7]. Language features also predict geographical [8–10] and temporal [11] variation in components of hedonic well-being such as positive and negative affect, and satisfaction with life.

In addition to state-of-the-art accuracy, LBAs appear to be face-valid, display reasonable test-retest reliability, and provide novel, theoretically relevant information about actual behaviors (e.g., personality traits [12]; well-being [13]), which is an important way to advance psychological science [14]. The accumulated evidence suggests that language is a behavioral manifestation of individual differences, including hedonic well-being, which can therefore be used to assess the latent variable of interest (but see [15,16] for a discussion of validity issues). Studies employing the Linguistic Inquiry and Word Count program (LIWC; [17]) have observed small but consistent associations between subjective well-being and language features (e.g., [5,18]). Building on the initial success of the LIWC’s “closed” vocabulary approach, recent “open-vocabulary” studies have identified novel associations between naturally occurring clusters of topics and individual differences [10]. In addition to evidencing greater predictive validity compared to the LIWC, topic clusters created through Latent Dirichlet Allocation (LDA; [19]) can be interpreted as behavioral expressions of individual difference constructs in the context of social media and can therefore suggest new hypotheses about the behavioral indicators of the construct.

Schwartz et al. [13] found that open-vocabulary LDA topic clusters offered significant incremental validity to the LIWC for predicting hedonic well-being. Additionally, significantly associated topic clusters referred to theoretically relevant topics and behaviors, such as references to professional employment, positive relationships, communal engagement, and positive engagement with activities. These results suggest that LBA methods are promising for the prediction of well-being constructs, as well as explaining their manifestation in social media contexts. However, LBA methods have primarily focused on hedonic well-being, and we consequently know little about whether other dimensions of well-being, such as eudaimonic well-being and psychological richness, can be assessed with alternative methods.

### Hedonic well-being, eudaimonic well-being, and psychological richness

Traditionally, theories of the good life have focused on eudaimonic well-being, which broadly refers to having purpose and meaning in life [20]. Eudaimonic well-being stems from an Aristotelian tradition regarding the optimal functioning of an individual, and is conceptually and empirically distinct from hedonic well-being. In contrast, hedonic well-being emphasizes a utilitarian approach to the degree of pleasure one experiences. More recent research suggests that the *psychologically rich life*, characterized by heterogeneous, novel, and interesting experiences [21], can be considered a distinct dimension of the good life [22]. A substantial minority of people across multiple cultures implicitly and explicitly idealize a psychologically rich life as their preferred form of the good life, compared to hedonic and eudaimonic well-being [23]. Psychological richness is psychometrically distinct from hedonic and eudaimonic well-being in both self-report and informant-report data [22], and the Psychologically Rich Life Questionnaire displays good discriminant and test-retest validity [24]. Additionally, informant reports of psychological richness were reliably associated with self-reports across two measurement periods, and the self-other agreement was comparable to the self-other agreement for constructs such as positive affect, negative affect, life satisfaction, and meaning in life [24], which indicates that trait-relevant cues about psychological richness are accessible to informants. This suggests that trait-relevant cues for psychological richness are likely to be present and retrievable from social media text data.

Evidence for a three-factor structure has been reported in comparisons of the Psychologically Rich Life Questionnaire with other well-validated measures of hedonic and eudaimonic well-being [24], as well as in the Good Life Scale (GLS), which is intended to simultaneously measure the three dimensions of well-being [22]. While psychological richness is positively associated with hedonic and eudaimonic well-being, novel experiences like studying abroad in another county are associated with group-level increases in psychological richness but not with increases in other well-being constructs ([25]; Studies 3 and 4). This evidence suggests that while many life experiences and outcomes have previously displayed null or negative relationships with hedonic or eudaimonic well-being, they might still be associated with unmeasured dimensions of well-being, such as psychological richness. Additionally, experience sampling data show that activities like taking short trips, engaging in artistic activities, or reporting a busy day were predictors of a psychologically rich day for college students, suggesting that the configuration of daily experiences associated with psychological richness is distinct from hedonic and eudaimonic well-being ([25]; Study 1).

### The present study

Because psychological richness is a novel well-being construct, there is no prior research investigating how well psychological richness can be predicted from social media text data, and how this precision compares to eudaimonic and hedonic well-being. In the present study, we first report on the internal structure validity, discriminant validity, and convergent validity of the Good Life Scale (GLS; [22]), which is intended to measure psychological richness, hedonic well-being, and eudaimonic well-being. We then test whether psychological richness, hedonic well-being, and eudaimonic well-being can be predicted from a large sample of Facebook text data by comparing the predictive validity of different approaches, such as using demographic data, LIWC topics, and LDA topics. We test the incremental predictive validity of linguistic features above and beyond demographic features for each of the GLS constructs, and compare the relative prediction accuracies between the GLS constructs to test whether psychological richness and eudaimonic well-being can be predicted as well as hedonic well-being.

Finally, the construct validity of psychological richness is still being established and evaluated. Because prior social media text mining investigations have found the LIWC and LDA topics associated with subjective well-being variables to be generally face valid [13], we also aim to describe and interpret the LIWC and LDA topics that are associated self-reports of psychological richness, hedonic well-being, and eudaimonic well-being. Our descriptive approach aims to identify language features that evidence the discriminant and face validity of psychological richness as a meaningful dimension of the good life. By describing the language features associated with psychological richness, we aim to identify novel linguistic-behavioral information about psychological richness that can inform future research into the nature of the construct.

## Method

### Ethics statement

The study procedures were approved by the Institutional Review Board of Purdue University (#IRB-2020–301); we obtained written informed consent.

### Participants and procedure

We paid for a targeted sample of 3,000 participants from the Qualtrics online survey panel. Data collection commenced on May 11th, 2020 and ended on June 15th, 2020. Participants had to be employed full-time, reside in the United States, be over the age of 18, and have a Facebook account with at least 500 words across all posts. To ensure that participants had sufficient words for language analysis, we obtained written informed consent to access their Facebook posts, collected their posts using the Facebook Graph API, and screened participants who had less than 500 words across all posts on their accounts (see [26] for the rationale behind this exclusion criteria). We obtained an initial sample of 3,215 participants who completed the survey. We retained an analytic sample of 2,644 participants after excluding cases for not having enough words, failing attention check questions, not completing the full GLS, and for not reporting data for gender, age, income, and education (69% female; median age = 43, range = 18–65). The 105 participants who selected “65 or older” were grouped in with the 27 participants who identified themselves as 65 in the initial survey. Additionally, two cases shared an identical Qualtrics participant ID, so we randomly removed one of the cases.

### Measures

#### Demographics.

We measured gender as a binary variable (male = 1, female = 2). We operationalized education as an ordinal variable (1 = *less than high school*, 2 = *high school diploma or equivalent*; 3 = *vocational or technical training*, 4 = *bachelor’s degree or equivalent*; 5 = *master’s degree or equivalent*, 6 = *doctoral degree*). Income was operationalized as a seven-point ordinal variable with varied increments (1 = *less than $10,000*, 2 = *$10,000–19,000*, 3 = *$20,000-$34,999*, 4 = *$35,000-$49,999*, 5 = *$50,000-$99,999*, 6 = *$100,000-$149,999*, 7 = *More than $150,000*).

#### Good Life Scale.

We used the Good Life Scale (GLS; [22]) to measure psychological richness alongside the well-established constructs of eudaimonic and hedonic well-being. Participants were asked to indicate the extent to which they agreed with 15 adjectives describing their lives (“My life has been…”) on a seven-point scale (1 = *strongly disagree*; 7 = *strongly agree*). The subscales are referred to as psychological richness (*interesting*, *dramatic*, *psychologically rich*, *uneventful*, and *monotonous*; *α* = .711), meaning (*meaningful*, *fulfilling*, *purposeful*, *meaningless*, and *disorganized*; *α* = .85), and happiness (*happy*, *enjoyable*, *comfortable*, *unstable*, and *sad*; *α* = .861) for brevity throughout the rest of the paper.

#### Big five.

We measured the Big Five trait domains of Openness (*M* = 5.1, *SD* = 1.21, *α* = .410), Conscientiousness (*M* = 5.54, *SD* = 1.22, *α* = .557), Extraversion (*M* = 3.88, *SD* = 1.58, *α* = .658), Agreeableness (*M* = 5.24, *SD* = 1.17, *α* = .386) and Neuroticism (*M* = 3.22, *SD* = 1.44, *α* = .704) with the Ten-Item Personality Inventory [27]. Items were presented on a seven-point scale (1 = *strongly disagree*; 7 = *strongly agree*). Scale reliabilities are low because each scale comprises just two items. While these internal reliability estimates may seem low, the two items for each Ten-Item Personality Inventory domain were intentionally selected to represent the breadth and heterogeneity of the domain.

### Analytic strategy

The design of the study and analyses were not pre-registered. These analyses are intended to be descriptive and exploratory, and are not designed to deductively test hypotheses.

We first conducted a three-factor and bi-factor confirmatory factor analysis of the GLS data with lavaan [28] package, and used the semTools [29] package to compute reliability statistics.

We incorporated both open-vocabulary and closed-vocabulary features — Latent Dirichlet Analysis (LDA; [19]) and Linguistic Inquiry and Word Count (LIWC; [30]) — to predict the Good Life Scale constructs. To represent users’ language, we employed LDA as the primary feature and generated 2000 topics using the DLATK package [31]. To ensure sufficient coverage and diversity, we used topics that were generated on larger datasets, which is a common practice in natural language processing. Specifically, we utilized 2000 English topics that were produced from a corpus of approximately 18 million Facebook updates [10]. To limit the number of topics per document, we set alpha to 0.30. Each topic is represented as a set of words with probabilities. We evaluated each post’s probability of containing each of the 2000 topics (*p*(topic, post)) by computing its probability of containing a word (*p*(word | post)) and the probability of the words being in given topics (*p*(topic | word)).

Furthermore, 73 English categories were procured from LIWC [30], which encompassed psychological, social, and syntactic categories. Relative frequency was computed for each dictionary, which was determined from the total number of times a word written by the user matched a word in each dictionary, divided by the user’s overall word count.

We assessed the Good Life Scale constructs with a variety of predictive models that relied on varying combinations of demographic variables, LDA topic data, and LIWC data as predictors. We used 10-fold cross-validation, a resampling technique that involves partitioning the dataset into complementary subsets, where a subset is used for testing while the remaining subsets are used for model training on multiple iterations. We tested incremental predictive validity between models with paired t-tests with the SciPy Python library [32]. To determine whether the accuracy of predictive models were significantly different between sub-scales, we conducted two-tailed z-tests for the difference between dependent correlations with an overlapping variable (in this case, the shared demographic and/or language feature predictors) with the *cocor* R package [33]. To examine the correlations between individual LDA topics and the GLS scales scores, we controlled for age and gender and applied the Benjamini–Hochberg correction for multiple testing [34].

#### Statistical power and effect size interpretation.

While a formal power analysis was not conducted a priori, a post-hoc power analysis with G*Power 3 ([35]; procedure: “exact test: correlation, bivariate normal model”) indicated that with our final analytic sample, we had 99% power to detect an effect size of *r* = .10 at *α* = .01 for a two-tailed test. A sensitivity power analysis indicated that we had 80% power to detect a correlation as low as *r* = .06 at *α* = .01 for a two-tailed test. We interpret an effect size of *r* = / > .20 as “medium”, *r* = / > .10 as “small”, and *r* = / > .05 as “very small” based on the effect size guidelines suggested by Funder and Ozer [36] for psychological research. We do not interpret effects smaller than this, as they are unlikely to be replicable or meaningful in this context. Therefore, we had high (99%) power to detect small effects, and adequate (80%) power to detect very small effects up to *r* = .06. Significant associations between individual LDA topics and responses to self-report measures of well-being are typically very small to small in prior research (e.g., [37]), and we are therefore adequately powered to observe associations between LDA topics and the GLS scores. One small effect in isolation may be the result of random noise (see [38]), however, a consistent and replicable pattern of small effects can be informative about the natural language used by people who perceive their lives to be happy, meaningful, or psychologically rich.

We interpret individual associations between LDA topics and GLS scores when the effect size is greater than or equal to the Funder and Ozer [36] “very small” criterion of *r* = .05 and has a (Benjamini–Hochberg corrected) *p*-value of <.01, as this threshold is tied to the replicability of an effect [39].

Following the suggestion of a reviewer, we also conducted a sensitivity analysis for the paired model comparison t-tests. We had 80% power to detect a difference as small as *d*_*z* _= .054 with our analytic sample of 2,644 at *α* = .05 for a two-tailed test. Therefore, we were adequately powered to detect very small differences between the models.

## Results

### Validity properties of the Good Life Scale

A confirmatory factor analysis of the Good Life Scale indicated that an orthogonal, three-factor solution was not a good fit for the data (χ^2^(90) = 6730.636, *p* < .001; CFI = .726; TLI = .680; RMSEA = .167; SRMR = .274) according to conventional standards [40]. The unidimensional $\omega_{u}$ reliability coefficients from the orthogonal CFA for psychological richness ($\omega_{u}$ =.72), meaning ($\omega_{u}$ =.85), and happiness ($\omega_{u}$ =.84) were similar to the *α* reliability coefficients mentioned in the Methods section. All ω reliability results are based on the omega2 estimator of $\omega_{u}$ in the semTools package, which uses the model-implied variance instead of the observed sample variance (see [41] for discussion of the differences between estimators). A bi-factor model that included a “general positivity” factor to account for the shared variance between the items moderately improved model fit (χ^2^(75) = 2926.320, *p* < .001; CFI = .882; TLI = .835; RMSEA = .120; SRMR = .078), but not to acceptable cutoffs apart from the SRMR. Factor loadings for the items in these models are included in Tables SA1 and SA2 of S1 File (https://osf.io/35nd2?view_only=020baa24c13f49ccb097405ea5b92cdf). Hierarchical $\omega_{h}$ reliability for the general factor was.80. There essentially no variance in the meaning subscale that was due to the specific factor above the general factor ($\omega_{h-ss}$ =.0004). Happiness had slightly more variance due to the specific factor ($\omega_{h-ss}$ =.27), and psychological richness had the most ($\omega_{h-ss}$ =.51).

We report the CFA results for transparency and context, but chose to refrain from modifying the scale further because the initial item pool was small, and we did not have a hold-out sample to test a modified model upon. Though the bi-factor model did indicate incrementally better fit, it also undermines the interpretability of the GLS for the LDA and prediction analyses that are the focus of the present study, so we use the unmodified GLS in the present research and encourage future scale development research to improve the psychometric properties of the GLS.

Psychological richness, meaning, and happiness all had significant, positive bivariate correlations with age, income, and education (Table 1). The positive association between psychological richness and income and education replicated prior meta-analytic estimates (Oishi & Westgate, 2021). Psychological richness and happiness were not significantly associated with gender, while meaning had a very small association (*r* = .06, *p* < .01).

**Table 1:** Means, standard deviations, and correlations between study variables.

Variable	*M*	*SD*	1	2	3	4	5	6
1. Psych. Richness	4.89	1.02						
2. Happiness	5.12	1.16	.19**					
			[.16,.23]					
3. Meaning	5.27	1.18	.45**	.71**				
			[.42,.48]	[.69,.73]				
4. Age	43.44	11.94	.12**	.11**	.17**			
			[.08,.15]	[.07,.15]	[.13,.20]			
5. Gender (Female)	1.70	0.46	.01	−.01	.06**	−.06**		
			[-.03,.05]	[-.05,.03]	[.02,.09]	[-.10, -.02]		
6. Income	4.44	1.20	.08**	.21**	.19**	.15**	−.17**	
			[.04,.12]	[.18,.25]	[.15,.22]	[.11,.18]	[-.21, -.14]	
7. Education	3.78	1.11	.08**	.15**	.15**	.02	−.03	.43**
			[.04,.12]	[.11,.19]	[.11,.19]	[-.02,.06]	[-.07,.00]	[.40,.46]

*Note.* Values in square brackets indicate the 95% confidence interval for each correlation. * *p* < .05. ** *p* < .01.

Finally, we assessed the discriminant and convergent validity of the Good Life Scale by examining the associations between each sub-scale and a brief measure of the Big Five. Psychological richness had the strongest association with Openness (*r* = .37, *p* < .01) relative to the meaning (*r* = .29, *p* < .01) and happiness (*r* = .17, *p* < .01) scales, which replicates prior research (Oishi & Westgate, 2021) and speaks to the convergent and discriminant validity of the psychological richness subscale. Psychological richness was also positively associated with Conscientiousness, Extraversion, and Agreeableness, and negatively associated with Neuroticism (see Table 2), which replicates prior meta-analytic findings [22]. However, the low reliability of the brief, two-item personality scales limits the precision of these results.

**Table 2:** Correlation between Good Life Scale and Big Five Traits.

	Openness	Conscientiousness	Extraversion	Agreeableness	Neuroticism
Psych. Richness	.37**	.15**	.29**	.18**	−.18**
	[.33,.40]	[.11,.19]	[.25,.32]	[.15,.22]	[-.21, -.14]
Happiness	.17**	.35**	.22**	.24**	−.51**
	[.13,.21]	[.32,.39]	[.18,.25]	[.20,.27]	[-.54, -.49]
Meaning	.29**	.49**	.25**	.32**	−.48**
	[.25,.32]	[.46,.52]	[.22,.29]	[.28,.35]	[-.51, -.45]

*Note.* Values in square brackets indicate the 95% confidence interval for each correlation. * *p* < .05. ** *p* < .01.

### Prediction of the Good Life Scale scores with demographic and language features

Predictive models for the GLS scales using seven combinations of language and demographic data are presented in Table 3. Prediction accuracy from demographic information alone was significantly higher for meaning (*z* = −6.0768, *p* < .001) and happiness (*z* = −3.9961, *p* < .001) compared to psychological richness, while prediction accuracy did not differ between meaning and happiness (*z* = −1.6846, *p* = .0921).

**Table 3:** Prediction of Good Life Scale Constructs (Pearson’s r).

		Psychological Richness	Happiness	Meaning
Demographic Predictors				
Model 1	Age + Gender	0.108	0.093	0.169
Model 2	Income + Education	0.086	0.219	0.197
Model 3	Age + Gender + Income + Education	0.134	0.231	0.255
Language Predictors				
Model 4	LIWC	0.184	0.270	0.291
Model 5	Topics	0.196	0.216	0.266
Model 6	LIWC + Topics	0.201	0.230	0.274
Demographic and Language Predictors				
Model 7	LIWC + Topics + Age + Gender + Income + Education	0.210	0.248	0.290

Prediction accuracy from linguistic features alone (Model 6) was not significantly different between psychological richness and happiness (*z* = −1.2164, *p* = 0.2238), but meaning was more accurate than psychological richness (*z* = −3.7424, *p* = 0.0002) and happiness (*z* = −3.1088, *p* = .0019). In the model that combined language features and demographics, the psychological richness model was still just as accurate as the happiness model (*z* = −1.5939, *p* = .111), but meaning was more accurate than psychological richness (*z* = −4.0897, *p* < .001) and happiness (*z* = −2.9569, *p* = .0031).

The addition of language features (i.e., LIWC and LDA) in Model 7 incrementally improved predictive accuracy over just using baseline demographic predictors (age, gender, income, and education in Model 3) for the prediction of psychological richness (*t*(2643) = −2.148, *p* = 0.016), but not for meaning (*t*(2643) = −1.530, *p* = 0.063) and happiness (*t*(2643) = −1.337, *p* = 0.091). Compared head-to-head, language features alone (Model 6) did not produce a significantly more accurate model compared to demographic features (Model 3) for psychological richness (*t*(2643) = −1.606, *p* = 0.054), happiness (*t*(2643) = −0.343, *p* = 0.366) and meaning (*t*(2643) = −0.637, *p* = 0.262). It should be noted that the most accurate model for happiness and meaning only used LIWC features, while the model that combined all language and demographic features was the most accurate for psychological richness.

### Language topic correlates of the Good Life Scale

Two of the authors (CVB and FZ) sorted the individual LDA topics that correlated at *p* < .01 with each of the factors into higher-order themes. All LDA associations that exceeded the *p*-value threshold also exceeded *r* > |.05| and were at least very small. Negative associations ranged from *r* = −.057 to −.210; positive associations ranged from *r* = .057 to.149. We only present themes with at least three example topics, and limit examples of each theme to the 3 largest effect sizes in the tables for brevity (see SB4 in S2 File for all topic correlations that correspond to each theme; https://osf.io/qanp3?view_only=020baa24c13f49ccb097405ea5b92cdf). We also present the closed-vocabulary LIWC category correlations, and note when the analogous LIWC categories conceptually replicated the results of the LDA analyses.

Topics positively correlated with the happiness factor (Table 5) were sorted into the labels of *Leisure and Entertainment* (e.g., *concert*, *shopping*, and *vacation*), *Family* (e.g., *family*, *son*, and *kiddos*), *Celebration and Gathering* (e.g., *birthday*, *thanksgiving*, and *anniversary*), *Gratitude* (e.g., *thanks*, *blessed*, and *grateful*), *God and Religion* (e.g., *church*, *worship*, and *god*), and *Education* (e.g., *college*, *degree*, and *graduation*). *Positive Affect* (e.g., amazing, awesome, fun) was also evident in many of the topics. Topics also referred to *Travel* (e.g., *flight*, *hotel*, *trip*) and *Geography* (e.g., *London*, *California*, *Tokyo*). The LIWC categories of Affiliation, Leisure, Positive Emotions, Family, and Religion were positively associated with happiness and conceptually replicate these results (Table 4). The LIWC categories of 1^st^ Person Plural Pronouns, Drives and Needs, Time, Home, Adjectives, and Work were also positively associated with happiness.

**Table 4:** LIWC categories significantly associated with happiness.

Topic	*r*	*p*	CI lower	CI upper
*Positive Associations*				
1st pers plural	0.138	0.000	0.100	0.175
Affiliation	0.136	0.000	0.098	0.173
Leisure	0.122	0.000	0.085	0.160
Positive emotion	0.110	0.000	0.072	0.148
Drives and Needs	0.090	0.000	0.052	0.128
Time	0.075	0.000	0.037	0.112
Family	0.064	0.002	0.026	0.102
Home	0.063	0.003	0.025	0.101
Religion	0.062	0.003	0.024	0.100
Adjectives	0.059	0.004	0.021	0.097
Work	0.054	0.009	0.016	0.092
*Negative Associations*				
Negative emotion	−0.233	0.000	−0.269	−0.197
Anger	−0.201	0.000	−0.237	−0.164
Negations	−0.185	0.000	−0.222	−0.148
Cognitive Processes	−0.174	0.000	−0.211	−0.137
Differentiation	−0.169	0.000	−0.205	−0.131
Tentative	−0.164	0.000	−0.201	−0.127
I	−0.156	0.000	−0.193	−0.118
Body	−0.154	0.000	−0.191	−0.117
Adverb	−0.150	0.000	−0.187	−0.112
Impersonal Pronouns	−0.147	0.000	−0.184	−0.109
Swear words	−0.146	0.000	−0.183	−0.109
Risk	−0.146	0.000	−0.183	−0.109
Total Pronouns	−0.146	0.000	−0.183	−0.108
Regular verbs	−0.140	0.000	−0.177	−0.102
Sadness	−0.135	0.000	−0.172	−0.097
Anxiety	−0.132	0.000	−0.169	−0.094
Interrogatives	−0.131	0.000	−0.169	−0.094
Auxiliary verbs	−0.127	0.000	−0.164	−0.089
Death	−0.126	0.000	−0.163	−0.088
Function Words	−0.125	0.000	−0.163	−0.088
Present Focus	−0.124	0.000	−0.162	−0.087
Personal Pronouns	−0.115	0.000	−0.153	−0.078
Health	−0.108	0.000	−0.146	−0.070
Fillers	−0.108	0.000	−0.146	−0.070
Sexual	−0.108	0.000	−0.146	−0.070
Discrepancy	−0.107	0.000	−0.144	−0.069
Cause	−0.105	0.000	−0.143	−0.068
Insight	−0.101	0.000	−0.138	−0.063
Informal Speech	−0.094	0.000	−0.132	−0.056
3rd Pers. Plural Pronouns	−0.089	0.000	−0.126	−0.051
Nonfluencies	−0.079	0.000	−0.116	−0.041
Conjunctions	−0.076	0.000	−0.114	−0.038
Biological Processes	−0.072	0.000	−0.110	−0.034
Past Focus	−0.070	0.001	−0.107	−0.031
Feeling	−0.069	0.001	−0.107	−0.031
Comparisons	−0.065	0.002	−0.103	−0.027

Topics negatively associated with happiness were described with the labels of *Negative Affect* (e.g., *anxiety*, *depressed*, and *scared*), *Hostility* (e.g., *stupid*, *crap*, and *hell*), *Discussion of Emotions/Mixed Emotions* (e.g., *feels, felt, laughing, crying*), *Body* (e.g., *head*, *eyes*, and *foot*), *Health* (e.g., *sick*, *flu*, and *infection*), *Communication* (e.g., *talking*, *asked*, and *calling*), *Money* (e.g., *tax, money, and bills*), *God and Religion* (e.g., *god, religion, praying*), *Slang* (e.g., *omg, cuz, lol*), *Questioning* (e.g., *why, questions, debating*), *Political and Social Issues* (e.g., *rights, politics, freedom*), and *Reflection and Insight* (e.g., *discovered, learned, lessons*). 36 LIWC categories were negative associated with happiness; for brevity we note that negative associations with Negative Emotion, Anger, Body, Swear, Sad, Anxious, Health, Informal Language, Nonfluencies, and Biological Processes conceptually replicate the LDA results. In terms of grammar, *Negations* (e.g., *haven’t*, *didn’t*, and *wasn’t*), 2^nd^ Person Pronouns (e.g., *you’re*, *you’ll*, and *you’ve*) and 3^rd^ Person (e.g., *they, they’re, themselves*) were negatively associated with happiness. The LIWC categories for Negations, Total Pronoun usage, and 2^nd^ Person pronouns were also negatively associated with happiness, but 3^rd^ Person singular and plural was not. There were also a non-trivial amount of essentially miscellaneous topics [31] that were not clearly interpretable in terms of a broader category, but still were significantly associated with happiness. We illustrate the content of the miscellaneous topics that had the highest correlations with happiness and meaning for context in Tables 5 and 6.

**Table 5:** Happiness topic correlations sorted by theme.

Theme	Topic Examples	*r*	*p*	CI lower	CI upper
**Positive Happiness Correlations**					
Leisure and Entertainment	great, had, friends, weekend, wonderful, food, such, lots, evening, company	0.149	0.000	0.112	0.186
	dinner, went, lunch, date, nice, shopping, hubby, evening, breakfast, afternoon	0.127	0.000	0.090	0.165
	fun, pool, park, kids, swimming, summer, water, swim, blast, afternoon	0.117	0.000	0.079	0.154
Family	happy, birthday, son, brother, boy, nephew, handsome, grandson, lil, oldest	0.120	0.000	0.082	0.157
	thanks, visit, wonderful, dad, aunt, uncle, enjoyed, cousin, cousins, visiting	0.114	0.000	0.076	0.152
	happy, birthday, anniversary, celebrating, wedding, husband, celebrate, dad, parents, 50th	0.114	0.000	0.077	0.152
	birthday, party, celebrating, cake, early, bday, dinner, present, celebrate, celebration	0.121	0.000	0.083	0.158
Celebration and Gathering	happy, birthday, son, brother, boy, nephew, handsome, grandson, lil, oldest	0.120	0.000	0.082	0.157
	party, parties, bday, graduation, halloween, dance, pool, tea, celebrate, invited	0.115	0.000	0.077	0.152
	great, had, friends, weekend, wonderful, food, such, lots, evening, company	0.149	0.000	0.112	0.186
Gratitude	thanks, visit, wonderful, dad, aunt, uncle, enjoyed, cousin, cousins, visiting	0.114	0.000	0.076	0.152
	beautiful, amazing, daughter, most, wonderful, such, absolutely, lovely, wife, lucky	0.098	0.000	0.060	0.136
	church, sunday, pastor, service, worship, baptist, bible, youth, group, ministry	0.100	0.000	0.062	0.137
God and Religion	god, bless, church, blessed, candace, blessing, service, pray, praise, blessings	0.075	0.000	0.037	0.113
	peace, blessings, joy, grace, faith, power, god’s, strength, presence, praise	0.073	0.001	0.035	0.111
	college, proud, university, students, student, award, congratulations, program, degree, graduate	0.117	0.000	0.079	0.154
Education	school, kids, camp, summer, kiddos, teacher, preschool, daycare, boot, kindergarten	0.102	0.000	0.064	0.140
	school, high, class, middle, senior, college, graduation, elementary, junior, reunion	0.090	0.000	0.052	0.128
	fun, had, much, having, blast, girls, lots, fair, tons, sooo	0.101	0.000	0.063	0.139
Positive Valence and Affect	an, amazing, awesome, such, job, incredible, experience, absolutely, truly, opportunity	0.100	0.000	0.063	0.138
	beautiful, amazing, daughter, most, wonderful, such, absolutely, lovely, wife, lucky	0.098	0.000	0.060	0.136
	flight, airport, plane, waiting, trip, fly, leaving, flying, delayed, chicago	0.095	0.000	0.058	0.133
Travel	hotel, tour, city, town, view, museum, tower, london, paris, building	0.094	0.000	0.056	0.132
	trip, road, vegas, vacation, travel, field, trips, las, booked, disney	0.078	0.000	0.040	0.115
	hotel, tour, city, town, view, museum, tower, london, paris, building	0.094	0.000	0.056	0.132
Geography	texas, trip, north, heading, nc, south, road, headed, tx, tn	0.072	0.001	0.034	0.110
	south, north, state, carolina, texas, virginia, dakota, states, florida, california	0.069	0.001	0.031	0.107
**Negative Happiness Correlations**					
Negative Affect	myself, alone, felt, feelings, knowing, lately, scared, anxiety, depressed, emotions	−0.206	0.000	−0.243	−0.170
	sometimes, cry, strong, hurt, tough, push, hurts, harder, gives, pull	−0.161	0.000	−0.198	−0.124
	health, mental, problem, problems, issues, anxiety, stress, depression, illness, physical	−0.152	0.000	−0.189	−0.115
	wrong, stupid, dumb, there’s, what’s, seriously, shame, smart, wtf, idiot	−0.210	0.000	−0.246	−0.173
Hostililty	damn, shit, pissed, wtf, seriously, hell, fucking, ugh, mind, omg	−0.200	0.000	−0.236	−0.163
	people, being, stop, stupid, tired, sick, seriously, drama, rude, crap	−0.199	0.000	−0.236	−0.162
Discussion of Emotions/Mixed Emotions	like, feel, makes, doesn’t, feels, felt, crap, seem, sound, guilty	−0.182	0.000	−0.218	−0.144
	feel, better, feeling, getting, myself, starting, feels, normal, slowly, felt	−0.130	0.000	−0.167	−0.092
	stop, laughing, crying, kid, sj, screaming, yelling, tears, literally, dying	−0.127	0.000	−0.164	−0.089
	eyes, hold, inside, hands, deep, stand, breath, feet, falling, holding	−0.144	0.000	−0.182	−0.107
Body	head, face, hand, nose, finger, mouth, cut, tongue, arm, foot	−0.127	0.000	−0.164	−0.089
	pain, knee, hurt, leg, foot, arm, hurts, ankle, neck, feet	−0.101	0.000	−0.139	−0.064
	health, mental, problem, problems, issues, anxiety, stress, depression, illness, physical	−0.152	0.000	−0.189	−0.115
Health	sick,:(, stomach, flu, feeling, poor, fever, cold, ugh, shot	−0.107	0.000	−0.145	−0.069
	pain, knee, hurt, leg, foot, arm, hurts, ankle, neck, feet	−0.101	0.000	−0.139	−0.064
	about, talking, thinking, talk, talked, worried, conversation, ralph, telling, waldo	−0.114	0.000	−0.152	−0.076
Communication	post, facebook, posting, posts, fb, comments, posted, picture, photo, pictures	−0.109	0.000	−0.147	−0.071
	said, asked, told, guy, called, wanted, looked, replied, boss, manager	−0.108	0.000	−0.145	−0.070
	were, didn’t, knew, wasn’t, felt, liked, looked, seemed, weren’t, hated	−0.159	0.000	−0.196	−0.122
Negation	yet, haven’t, even, i’ve, since, already, though, hasn’t, seen, gotten	−0.149	0.000	−0.186	−0.112
	didn’t, wasn’t, wanted, couldn’t, tried, wouldn’t, decided, knew, realize, turned	−0.149	0.000	−0.186	−0.111
	i’ll, you’re, cause, wanna, won’t, you’ll, ain’t, there’s, alone, you’ve	−0.182	0.000	−0.219	−0.145
2nd person	always, you’re, something, you’ll, wrong, there’s, you’ve, yourself, what’s, happens	−0.163	0.000	−0.200	−0.125
	when, you’re, moment, happens, realize, you’ve, comes, welcome, means, they’re	−0.139	0.000	−0.176	−0.101
	pay, money, attention, paid, bills, paying, bill, owe, rent, afford	−0.126	0.000	−0.163	−0.088
Money	tax, pay, money, taxes, insurance, government, dollars, income, jobs, health	−0.064	0.006	−0.102	−0.026
	free, gift, earn, use, money, points, link, cash, cards, sign	−0.059	0.007	−0.097	−0.021
3rd Person Plural/Interpersonal Topics	others, yourself, judge, themselves, hurt, actions, respect, feelings, opinion, situation	−0.182	0.000	−0.219	−0.145
	they, people, why, understand, dont, ppl, wonder, act, they’re, realize	−0.174	0.000	−0.211	−0.137
	someone, person, yourself, kind, others, respect, kindness, trust, treat, relationship	−0.147	0.000	−0.184	−0.110
	god, us, faith, trust, plan, god’s, bible, lives, prayer, control	−0.133	0.000	−0.170	−0.096
God and Religion	country, christian, religion, against, freedom, agree, religious, america, christians, liberal	−0.083	0.000	−0.121	−0.046
	better, feeling, hope, hoping, feel, soon, hopefully, goes, gets, praying	−0.074	0.001	−0.112	−0.036
	lol, im, lmao, haha, omg, crazy, wow, thats, wtf, dude	−0.118	0.000	−0.155	−0.080
Slang	ur, dont, im, yu, ppl, shit, cuz, aint, cant, thats	−0.107	0.000	−0.144	−0.069
	ha, lol, guess, haha, couldn’t,:p, sorry,;), resist, yep	−0.071	0.001	−0.109	−0.033
	why, does, wondering, wonder, understand, reason, wonders, explain, reasons, seem	−0.136	0.000	−0.173	−0.098
Questioning	question, answer, questions, ask, response, answered, following, below, asked, wrong	−0.091	0.000	−0.129	−0.054
	or, either, whether, decide, maybe, both, whatever, question, decisions, debating	−0.057	0.009	−0.095	−0.019
	women, men, gay, sex, woman, female, male, sexual, rights, marriage	−0.132	0.000	−0.170	−0.095
Political and Social Issues	political, society, human, social, culture, media, politics, common, based, opinion	−0.090	0.000	−0.127	−0.052
	country, christian, religion, against, freedom, agree, religious, america, christians, liberal	−0.083	0.000	−0.121	−0.046
	were, became, lived, known, several, began, ended, recently, discovered, grew	−0.107	0.000	−0.145	−0.069
Reflection and Insight	life, learned, learn, lesson, lessons, taught, learning, grateful, journey, mistakes	−0.078	0.000	−0.115	−0.040
	life, world, whole, real, living, makes, worth, simple, important, wouldn’t	−0.059	0.009	−0.097	−0.021
	when, someone, tell, ask, they’re, happens, tells, somebody, expect, asks	−0.187	0.000	−0.224	−0.150
Miscellaneous	guy, kind, they’re, i’d, probably, wasn’t, joke, weird, jeopardy, you’d	−0.181	0.000	−0.217	−0.143
	do, can, nothing, else, anything, everything, doing, wrong, absolutely, nobody	−0.159	0.000	−0.196	−0.122

**Table 6:** Meaning topic correlations sorted by theme.

Theme	Topic Examples	*r*	*p*	CI lower	CI upper
**Positive Meaning Correlations**					
Education	College, proud, university, students, student, award, congratulations, program, degree, graduate	0.114	0.000	0.076	0.151
	School, kids, camp, summer, kiddos, teacher, preschool, daycare, boot, kindergarten	0.101	0.000	0.064	0.139
	School, high, class, middle, senior, college, graduation, elementary, junior, reunion	0.101	0.000	0.063	0.138
	Am, thankful, family, blessed, wonderful, grateful, thanksgiving, truly, husband, lucky	0.130	0.000	0.093	0.168
Gratitude	Beautiful, amazing, daughter, most, wonderful, such, absolutely, lovely, wife, lucky	0.124	0.000	0.086	0.161
	Thanks, visit, wonderful, dad, aunt, uncle, enjoyed, cousin, cousins, visiting	0.116	0.000	0.078	0.153
Positive Valence and Affect	Am, thankful, family, blessed, wonderful, grateful, thanksgiving, truly, husband, lucky	0.130	0.000	0.093	0.168
	Great, had, friends, weekend, wonderful, food, such, lots, evening, company	0.127	0.000	0.090	0.165
	Beautiful, amazing, daughter, most, wonderful, such, absolutely, lovely, wife, lucky	0.124	0.000	0.086	0.161
	Church, sunday, pastor, service, worship, baptist, bible, youth, group, ministry	0.119	0.000	0.081	0.156
God and Religion	Peace, blessings, joy, grace, faith, power, god’s, strength, presence, praise	0.109	0.000	0.071	0.147
	God, bless, church, blessed, candace, blessing, service, pray, praise, blessings	0.102	0.000	0.064	0.139
	Am, thankful, family, blessed, wonderful, grateful, thanksgiving, truly, husband, lucky	0.130	0.000	0.093	0.168
Family	Beautiful, amazing, daughter, most, wonderful, such, absolutely, lovely, wife, lucky	0.124	0.000	0.086	0.161
	Thanks, visit, wonderful, dad, aunt, uncle, enjoyed, cousin, cousins, visiting	0.116	0.000	0.078	0.153
	Happy, birthday, son, brother, boy, nephew, handsome, grandson, lil, oldest	0.115	0.000	0.078	0.153
Celebration and Gathering	Happy, birthday, girl, baby, beautiful, proud, sweet, ago, young, joy	0.113	0.000	0.075	0.150
	Happy, birthday, anniversary, celebrating, wedding, husband, celebrate, dad, parents, 50th	0.104	0.000	0.066	0.142
	Great, had, friends, weekend, wonderful, food, such, lots, evening, company	0.127	0.000	0.090	0.165
Leisure and Entertainment	Dinner, went, lunch, date, nice, shopping, hubby, evening, breakfast, afternoon	0.091	0.000	0.053	0.128
	Fun, pool, park, kids, swimming, summer, water, swim, blast, afternoon	0.085	0.000	0.047	0.123
	Texas, trip, north, heading, nc, south, road, headed, tx, tn	0.069	0.002	0.031	0.107
Geography	Hotel, tour, city, town, view, museum, tower, london, paris, building	0.057	0.009	0.019	0.095
	New, york, orleans, brand, city, jersey, beginning, england, chapter, adventure	0.057	0.009	0.019	0.095
**Negative Meaning Correlations**					
Hostility	Wrong, stupid, dumb, there’s, what’s, seriously, shame, smart, wtf, idiot	−0.207	0.000	−0.243	−0.170
	People, being, stop, stupid, tired, sick, seriously, drama, rude, crap	−0.185	0.000	−0.222	−0.148
	Damn, shit, pissed, wtf, seriously, hell, fucking, ugh, mind, omg	−0.175	0.000	−0.212	−0.138
	Myself, alone, felt, feelings, knowing, lately, scared, anxiety, depressed, emotions	−0.147	0.000	−0.184	−0.109
Negative Affect	I’m, sure, thinking, glad, tired, scared, nervous, afraid, kinda, confused	−0.143	0.000	−0.180	−0.105
	Now, right, left, confused, frustrated, rite, hating, pretend, liking, stressed	−0.123	0.000	−0.160	−0.085
	Yet, haven’t, even, i’ve, since, already, though, hasn’t, seen, gotten	−0.174	0.000	−0.211	−0.137
Negation	Didn’t, wasn’t, wanted, couldn’t, tried, wouldn’t, decided, knew, realize, turned	−0.155	0.000	−0.192	−0.117
	Were, didn’t, knew, wasn’t, felt, liked, looked, seemed, weren’t, hated	−0.141	0.000	−0.178	−0.103
	Dr, doctor, appointment, office, waiting, doctors, appt, dentist, doctor’s, doc	−0.130	0.000	−0.168	−0.093
Health	Sick,:(, stomach, flu, feeling, poor, fever, cold, ugh, shot	−0.097	0.000	−0.134	−0.059
	Disease, pain, struggle, ms, inside, fine, arthritis, lupus, daily, diabetes	−0.080	0.000	−0.117	−0.042
	Movie, film, movies, story, watched, disney, original, scene, films, action	−0.089	0.000	−0.127	−0.051
Leisure and Entertainment	Game, playing, play, games, wii, video, mario, bowling, super, xbox	−0.081	0.000	−0.119	−0.043
	Dead, zombie, total, evil, ghost, halloween, american, horror, witch, scary	−0.080	0.000	−0.118	−0.042
Discussion of Emotions/Mixed Emotions	Like, feel, makes, doesn’t, feels, felt, crap, seem, sound, guilty	−0.183	0.000	−0.220	−0.146
	Good, bad, news, thing, times, pretty, idea, feels, mood, terrible	−0.104	0.000	−0.141	−0.066
	Feel, better, feeling, getting, myself, starting, feels, normal, slowly, felt	−0.100	0.000	−0.138	−0.063
	Car, parking, lot, street, front, hit, road, truck, saw, side	−0.091	0.000	−0.128	−0.053
Transportation	Car, home, drive, truck, stuck, ride, thru, driveway, driving, drove	−0.074	0.000	−0.112	−0.036
	Driving, car, drive, driver, license, bus, drivers, speed, road, turn	−0.073	0.001	−0.111	−0.035
	Gonna, sleep, guess, bed, i’ll, im, try, gotta, ill, hopefully	−0.107	0.000	−0.144	−0.069
Sleep	Sleep, asleep, awake, fall, bed, tired, wake, fell, wide, sleeping	−0.064	0.003	−0.102	−0.026
	Hours, sleep, 24, hour, 12, hrs, shift, spent, 1/2, slept	−0.058	0.009	−0.096	−0.020
	Head, face, hand, nose, finger, mouth, cut, tongue, arm, foot	−0.122	0.000	−0.159	−0.084
Body	Eyes, hold, inside, hands, deep, stand, breath, feet, falling, holding	−0.108	0.000	−0.146	−0.071
	Pain, knee, hurt, leg, foot, arm, hurts, ankle, neck, feet	−0.075	0.000	−0.113	−0.037
	Ur, dont, im, yu, ppl, shit, cuz, aint, cant, thats	−0.119	0.000	−0.156	−0.081
Slang	Lol, im, lmao, haha, omg, crazy, wow, thats, wtf, dude	−0.098	0.000	−0.135	−0.060
	Ya, y’all, ain’t, hey, gotta, em, smh, bout, yo, cuz	−0.087	0.000	−0.125	−0.049
	About, talking, thinking, talk, talked, worried, conversation, ralph, telling, waldo	−0.102	0.000	−0.140	−0.065
Communication	Phone, call, number, text, cell, calls, numbers, message, calling, answer	−0.096	0.000	−0.134	−0.059
	Said, asked, told, guy, called, wanted, looked, replied, boss, manager	−0.095	0.000	−0.133	−0.057
3rd Person Plural/Interpersonal Topics	They, people, why, understand, dont, ppl, wonder, act, they’re, realize	−0.154	0.000	−0.191	−0.116
	Someone, person, yourself, kind, others, respect, kindness, trust, treat, relationship	−0.133	0.000	−0.170	−0.095
	Others, yourself, judge, themselves, hurt, actions, respect, feelings, opinion, situation	−0.129	0.000	−0.166	−0.091
	I’ll, you’re, cause, wanna, won’t, you’ll, ain’t, there’s, alone, you’ve	−0.162	0.000	−0.199	−0.125
2nd person	Always, you’re, something, you’ll, wrong, there’s, you’ve, yourself, what’s, happens	−0.151	0.000	−0.188	−0.113
	When, you’re, moment, happens, realize, you’ve, comes, welcome, means, they’re	−0.136	0.000	−0.173	−0.098
	Guy, kind, they’re, i’d, probably, wasn’t, joke, weird, jeopardy, you’d	−0.201	0.000	−0.237	−0.164
Miscellaneous	When, someone, tell, ask, they’re, happens, tells, somebody, expect, asks	−0.173	0.000	−0.209	−0.135
	Do, would, something, anything, i’d, letter, normally, nope, favor, minds	−0.159	0.000	−0.196	−0.122

All of the topics positively associated with the meaning subscale (Table 6) were redundant with the themes identified in the happiness factor: *Leisure and Entertainment, Family, Celebration and Gathering, Gratitude, God and Religion, Education, Positive Affect, Travel, and Geography.* The LIWC categories that were positively associated with meaning were also redundant with the happiness associations (Table 7).

**Table 7:** LIWC categories significantly associated with meaning.

Topic	*r*	*p*	CI lower	CI upper
*Positive Associations*				
Affiliation	0.169	0.000	0.131	0.206
1st person plural	0.158	0.000	0.120	0.194
Positive emotion	0.119	0.000	0.081	0.156
Drives and Needs	0.108	0.000	0.070	0.145
Religion	0.097	0.000	0.059	0.134
Family	0.094	0.000	0.056	0.132
Leisure	0.070	0.000	0.032	0.108
Work	0.063	0.002	0.025	0.101
Time	0.061	0.003	0.023	0.099
Home	0.059	0.006	0.021	0.097
Common adjectives	0.057	0.006	0.018	0.094
*Negative Associations*				
Negative emotion	−0.194	0.000	−0.231	−0.157
Negations	−0.187	0.000	−0.224	−0.150
Anger	−0.186	0.000	−0.223	−0.149
Tentative	−0.158	0.000	−0.195	−0.121
Differentiation	−0.155	0.000	−0.192	−0.118
Cognitive processes	−0.148	0.000	−0.185	−0.111
Adverbs	−0.143	0.000	−0.180	−0.106
Swear words	−0.128	0.000	−0.166	−0.091
Regular verbs	−0.125	0.000	−0.163	−0.088
Death	−0.122	0.000	−0.159	−0.084
Risk	−0.121	0.000	−0.158	−0.083
Fillers	−0.114	0.000	−0.151	−0.076
Impersonal pronouns	−0.111	0.000	−0.148	−0.073
Body	−0.109	0.000	−0.146	−0.071
1st person singular	−0.108	0.000	−0.146	−0.070
Present focus	−0.106	0.000	−0.144	−0.068
Auxiliary verbs	−0.103	0.000	−0.141	−0.065
Interrogatives	−0.100	0.000	−0.138	−0.063
Discrepancies	−0.094	0.000	−0.132	−0.056
Sexuality	−0.089	0.000	−0.127	−0.051
Informal Speech	−0.089	0.000	−0.126	−0.051
Sadness	−0.084	0.000	−0.122	−0.046
Nonfluencies	−0.081	0.000	−0.119	−0.043
Total pronouns	−0.080	0.000	−0.118	−0.042
Insight	−0.080	0.000	−0.117	−0.042
Past focus	−0.078	0.000	−0.116	−0.040
Anxiety	−0.078	0.000	−0.115	−0.040
Function Words	−0.078	0.000	−0.115	−0.040
Comparatives	−0.077	0.000	−0.115	−0.039
Cause	−0.074	0.000	−0.111	−0.036
3rd Person Plural	−0.071	0.001	−0.109	−0.033

Like the happiness factor, many of the negative topic correlations with meaning could be labeled under the themes of *Negative Affect*, *Hostility*, *Health*, *Body, Slang, Discussion of Emotions/Mixed Emotions,* and *Negation*. Additionally, we categorized topics dealing with *Sleep* (e.g., *slept*, *bed*, and *tired*), *Transportation* (e.g., *drive*, *car*, and *bus*), and *Communication* (e.g., *why*, *wondering*, and *question*). Notably, several *Leisure and Entertainment* (e.g., *comic*, *disney*, and *movie*) topics were also negatively correlated with meaning. The use of 3^rd^ Person Plural Pronouns and 2^nd^ Person Pronouns was also negatively associated with meaning. The LIWC Total Pronouns and 3^rd^ Person Plural categories were also negatively associated with meaning, but the 2^nd^ Person category was not. All of the LIWC categories negatively associated with meaning were also negatively associated with happiness.

We synthesized the positive topic correlations with psychological richness into the themes of *Reflection and Insight* (e.g.*, discovered*, *learned*, and *lesson*), *Political and Social Issues*, *Negative Affect*, *God and Religion*, *Interpersonal Topics* (e.g., *others, human,* and *families*), and *Ideas and Concepts* (e.g., *intelligent, creative, thoughts,* and *research*) (Table 8). It is notable that topics mentioning *Political and Social Issues* were positively associated with psychological richness, while the same topics were typically negatively associated with happiness. It is also notable that topics mentioning *Negative Affect* were positively associated with psychological richness, while the same topics were typically negatively associated with happiness and meaning. The LIWC category of Anxiety was also positively associated with psychological richness (*r* = .067, *p* = .006), verifying this observation. *Ideas and Concepts* was a unique theme that characterized topic correlations that did not occur with the other constructs.

**Table 8:** Psychological richness topic correlations sorted by theme.

Theme	Topic Examples	*r*	*p*	CI lower	CI upper
**Positive Psych Richness Correlations**					
Interpersonal Topics	mind, others, human, yourself, become, peace, happiness, thoughts, joy, ourselves	0.136	0.000	0.099	0.174
	each, others, together, lives, important, families, stories, ways, shared, difference	0.132	0.000	0.095	0.169
	please, help, share, sign, dear, someone, stop, save, petition, somebody	0.108	0.000	0.070	0.146
	personality, strong, intelligent, creative, beauty, smart, unique, perfectly, honest, deep	0.138	0.000	0.100	0.175
Ideas and Concepts	mind, others, human, yourself, become, peace, happiness, thoughts, joy, ourselves	0.136	0.000	0.099	0.174
	political, society, human, social, culture, media, politics, common, based, opinion	0.121	0.000	0.083	0.158
	each, others, together, lives, important, families, stories, ways, shared, difference	0.132	0.000	0.095	0.169
Reflection and Insight	life, learned, learn, lesson, lessons, taught, learning, grateful, journey, mistakes	0.124	0.000	0.086	0.161
	life, world, whole, real, living, makes, worth, simple, important, wouldn’t	0.097	0.000	0.059	0.135
	political, society, human, social, culture, media, politics, common, based, opinion	0.121	0.000	0.083	0.158
Political and Social Issues	women, men, gay, sex, woman, female, male, sexual, rights, marriage	0.091	0.000	0.053	0.128
	country, christian, religion, against, freedom, agree, religious, america, christians, liberal	0.082	0.001	0.044	0.119
	about, say, something, worry, talk, care, complain, talking, worried, complaining	0.115	0.000	0.077	0.153
Negative Affect	heart, broken, tears, soul, pain, eyes, breaks, joy, hurt, attack	0.102	0.000	0.064	0.140
	health, mental, problem, problems, issues, anxiety, stress, depression, illness, physical	0.092	0.000	0.054	0.129
God and Religion	god, spirit, which, christ, lord, jesus, spiritual, holy, shall, unto	0.083	0.001	0.045	0.120
	country, christian, religion, against, freedom, agree, religious, america, christians, liberal	0.082	0.001	0.044	0.119
	him, god, reflection, jesus, word, god’s, spirit, faith, pray, relationship	0.081	0.001	0.043	0.119
**Negative Psych Richness Correlations**					
Leisure and Entertainment	game, playing, play, games, wii, video, mario, bowling, super, xbox	−0.089	0.000	−0.127	−0.051
	game, soccer, football, team, baseball, play, season, practice, basketball, games	−0.089	0.000	−0.126	−0.051
	game, win, hockey, team, usa, cup, vs, nba, soccer, won	−0.082	0.001	−0.119	−0.044

These divergent results provide some ecologically valid support for the notion that the construct of psychological richness may characterize thoughts, feelings, and behaviors that are substantially distinct from the other facets of the good life. The LIWC categories of Conjunctions, Health, Hearing, Total Pronouns, Personal Pronouns, Certainty, and Interrogatives were also positively associated with psychological richness (Table 9).

**Table 9:** LIWC categories significantly associated with psychological richness.

Topic	*r*	*p*	CI lower	CI upper
Conjunctions	0.104	0.000	0.066	0.142
Health	0.082	0.001	0.044	0.120
Hearing	0.078	0.001	0.040	0.116
Total pronouns	0.072	0.005	0.034	0.110
Personal pronouns	0.070	0.005	0.032	0.108
Certainty	0.067	0.005	0.029	0.105
Anxiety	0.067	0.006	0.029	0.105
Interrogatives	0.065	0.007	0.027	0.103
Leisure	−0.069	0.005	−0.107	−0.031

Among the negative correlations, the only theme that characterized three or more topics was *Leisure and Entertainment* (e.g., *xbox*, *football*, and *movie*). This was verified by a negative correlation (*r* = −.069, *p* = .005) with the LIWC Leisure topic. The individual *Leisure and Entertainment* topics for psychological richness had positive or null relationships with happiness, and null relationships with meaning (with the exception of one negative meaning association; see SB4 in S2 File).

## Discussion

In the present research, we found that language-based assessments of social media text data only added significant incremental predictive validity above and beyond demographic features for the prediction of psychological richness, but not for hedonic well-being or eudaimonic well-being. At the same time, psychological richness had the smallest relative predictability in all models and had notably fewer LIWC and LDA topic correlations compared to the other constructs. These data suggest that while language features can aid in the prediction of psychological richness, the overall predictability is still relatively low compared to other well-being constructs. One explanation for this result is that psychological richness is relatively low in observability and produces fewer trait-relevant cues that can be utilized by observers [42]. However, because previous research indicates that trait-relevant cues are available to inform accurate informant reports [24], it may also be the case that these cues are less recoverable with social media text data. The use of social media may not provide as many opportunities for people to behave in ways that are reflective of their standing on self-perceived psychological richness compared to hedonic and eudaimonic well-being. General aspects of using social media — or specific aspects of the Facebook platform [15] — may create an environment that encourages people to disclose positive experiences, while sharing more complex and ambiguous experiences is less common. Features of the platform may make psychological richness particularly hard to observe, whereas it may be more observable from text data generated in other environments and contexts.

The prediction coefficients for language-only models were all below the meta-analytic *r* of.33 for the prediction of individual-level well-being scores from social media text data with similar open and closed vocabulary methods [43]. A number of factors, such as the nature of the sample, the psychometric properties of the Good Life Scale instrument, and the nature of the trait’s observability and expression in the social media platform context, potentially contributed to the prediction performance.

Diener and Seligman [2] emphasized the importance of measuring different components of well-being beyond life satisfaction so that decision-makers can have a fully informed understanding of population-level well-being outcomes. Indeed, more governments and intergovernmental organizations recognize the need to supplement traditional economic indicators with well-being indicators [44]. Yet, the use of population surveys to obtain well-being indices is costly and time-consuming. From a population perspective, the use of social media text mining can potentially provide a real-time approach to assess distinct and established components of well-being in the population. This can serve to guide policy decisions as policymakers seek to incorporate and evaluate policy impact through well-being outcomes [45]. While the prediction coefficients for language-only and demographics-only models were similar, the added value of social media text data was only clear in the case of psychological richness, which was also the least predictable within each model compared to happiness and meaning. Advances in both machine learning and accurate psychological measurement are likely required for large-scale, passive assessments to reach their full potential. It may be that while individual-level prediction is still limited, regional prediction of well-being levels will soon be accurate enough to begin to inform policy decisions (meta-analytic *r* = .54; [43].

Another aim of this research was to learn more about the everyday behaviors that are associated with psychological richness, happiness, and meaning. The associations between the LDA topics and the GLS scales provide content and discriminant validity evidence for the language-based assessment of these well-being constructs, which is an essential step in validating a novel technology-based assessment method [46]. The association between psychological richness and the *Reflection and Insight, Ideas and Concepts, Political and Social Issues,* and *Negative Affect* topics can be interpreted as indicators of the processing of emotions, experiences, and ideas, which is consistent with the notion that psychological richness involves complex — but not necessarily enjoyable — experiences. Some of the *Negative Affect* and *Political and Social Issues* topics were positively associated with psychological richness while being negatively associated with happiness, which provided some discriminant validity evidence based on linguistic behavior. The only pattern among the topics that negatively correlated with psychological richness was that many referenced *Leisure and Entertainment*. The content in these topics referenced a variety of entertainment and leisure activities that are commonly understood as entertaining or enjoyable, and were positively associated with the happiness scale. Perhaps people who perceive their lives as less psychologically rich and interesting are more likely to be immersed in simple, familiar pleasures like playing video games or watching sports and movies. The effect sizes were incredibly small, however, and further multi-method evidence is needed to verify this observation.

The topics positively correlated with the happiness factor were face valid and in line with previous research on language-based prediction of life satisfaction [13]. The *Family* and *Celebration and Gathering* topics can be interpreted in terms of the extensive literature on the positive association between social connection and well-being [47], while the *God and Religion* topics can be interpreted in reference to the literature on the positive correlation between religiosity/spirituality and well-being [48]. While many of the negative topic correlations with happiness and meaning were face valid (e.g., *Negative Affect*, *Hostility*, *Health*, *Body*, *Discussion of Emotions*) in reference to lay definitions of the constructs, there were also themes that were less straightforwardly interpretable (e.g., *Time*, *Sleep*, *Money*, *Transportation*, *Communication*, *Questioning*), or had primarily grammatical content (e.g., *Negation*, *Slang*, 2^nd^ Person and 3^rd^ Person Pronouns).

The substantial content overlap between the topics positively associated with the happiness and meaning factors can be most straightforwardly explained by the strong, positive correlation between the scales (*r* = .71) and the lack of good fit in the confirmatory factor analysis. A major limitation of the present research was the poor model fit of the Good Life Scale. Future research should investigate whether the predictability of distinct well-being constructs from social media text data differs for instruments with better-differentiated multidimensionality. These results highlight the importance of measurement model fit and item content in determining the predictability from language, and interpretability of the associated language features. Future research that operationalizes the constructs of hedonic life satisfaction, eudaimonic well-being, and psychological richness with more comprehensive and distinct measures may replicate some of our results but is likely to differ in meaningful ways.

## Conclusion

We found that the recently proposed third component of well-being, psychological richness, could be predicted through language-based assessment methods, and that social media text data incrementally improved the prediction accuracy. However, the measures of happiness and meaning were just as predictable from demographics as they were from social media text data. These results highlight the central role that internal structure validity and item content may play in language-based assessments of psychological tests. Our results also provide additional discriminant validity evidence for the distinctiveness of the psychological richness construct by illustrating how its associations with everyday behaviors differed from the associations for the happiness and meaning scales. Future work should examine the degree to which the accuracy of LBA assessment of distinct components of well-being can be improved. Assessments with improved accuracy and discriminant validity may eventually be useful for understanding how distinct components of well-being are associated with policy changes and socio-economic conditions over time.

## Supporting information

S1 File Table SA1. Factor Loadings for Orthogonal 3-factor CFA of the Good Life Scale. Table SA2. Factor Loadings for Orthogonal Bifactor CFA of the Good Life Scale.
(DOCX)

S2 File Table SB1. LIWC Correlations. Table SB2. LDA Correlations. Table SB3. T-test Model Comparisons. Table SB4. All LDA Themes. Table SB5. Abbreviated LDA Themes.
(XLSX)
