# Supplementary material for: The assessment of psychological richness, meaning, and happiness with social media text data: Predictive accuracy and distinct behavioral correlates
Source: PLoS One. 2026 Jan 7;21(1):e0337649. doi: 10.1371/journal.pone.0337649 (PMC12779146; doi:10.1371/journal.pone.0337649)
Supplement: S1 File — Table SA1. Factor Loadings for Orthogonal 3-factor CFA of the Good Life Scale. Table SA2. Factor Loadings for Orthogonal Bifactor CFA of the Good Life Scale. (DOCX) [file pone.0337649.s001.docx]

**Supplemental Results A:**

**Confirmatory Factor Analysis Results for Good Life Scale**

Table SA1.

*Factor Loadings for Orthogonal 3-factor CFA of the Good Life Scale*

| Item | Psych Richness | Happiness | Meaning |
| --- | --- | --- | --- |
| 1. interesting | 0.706 | 0 | 0 |
| 2. dramatic | 0.348 | 0 | 0 |
| 3. psychologically rich | 0.516 | 0 | 0 |
| 4. uneventful (R) | 0.678 | 0 | 0 |
| 5. monotonous (R) | 0.674 | 0 | 0 |
| 6. happy | 0 | 0.915 | 0 |
| 7. enjoyable | 0 | 0.926 | 0 |
| 8. comfortable | 0 | 0.742 | 0 |
| 9. unstable (R) | 0 | 0.527 | 0 |
| 10. sad (R) | 0 | 0.607 | 0 |
| 11. meaningful | 0 | 0 | 0.881 |
| 12. fulfilling | 0 | 0 | 0.918 |
| 13. purposeful | 0 | 0 | 0.907 |
| 14. meaningless (R) | 0 | 0 | 0.637 |
| 15. disorganized (R) | 0 | 0 | 0.393 |

Table SA2.

*Factor Loadings for Orthogonal Bifactor CFA of the Good Life Scale*

| Item | General Factor | Psych Richness | Happiness | Meaning |
| --- | --- | --- | --- | --- |
| 1. interesting | 0.559 | 0.465 | 0 | 0 |
| 2. dramatic | -0.026 | 0.449 | 0 | 0 |
| 3. psychologically rich | 0.474 | 0.275 | 0 | 0 |
| 4. uneventful (R) | 0.273 | 0.682 | 0 | 0 |
| 5. monotonous (R) | 0.416 | 0.533 | 0 | 0 |
| 6. happy | 0.741 | 0 | 0.537 | 0 |
| 7. enjoyable | 0.764 | 0 | 0.526 | 0 |
| 8. comfortable | 0.576 | 0 | 0.469 | 0 |
| 9. unstable (R) | 0.437 | 0 | 0.290 | 0 |
| 10. sad (R) | 0.535 | 0 | 0.293 | 0 |
| 11. meaningful | 0.833 | 0 | 0 | 0.306 |
| 12. fulfilling | 0.895 | 0 | 0 | 0.232 |
| 13. purposeful | 0.863 | 0 | 0 | 0.245 |
| 14. meaningless (R) | 0.701 | 0 | 0 | -0.146 |
| 15. disorganized (R) | 0.512 | 0 | 0 | -0.404 |
